# Venom of Parasitoid *Pteromalus puparum* Impairs Host Humoral Antimicrobial Activity by Decreasing Host Cecropin and Lysozyme Gene Expression

**DOI:** 10.3390/toxins8020052

**Published:** 2016-02-20

**Authors:** Qi Fang, Bei-Bei Wang, Xin-Hai Ye, Fei Wang, Gong-Yin Ye

**Affiliations:** 1 State Key Laboratory of Rice Biology & Key Laboratory of Agricultural Entomology of Ministry of Agriculture, Institute of Insect Sciences, Zhejiang University, Hangzhou 310058, Zhejiang, China; fangqi@zju.edu.cn (Q.F.); wangbei_zju@163.com (B.-B.W.); wf87670701@163.com (F.W.); 2 College of Agriculture, South China Agricultural University, Guangzhou 510642, Guangdong, China; yexinhai1204@hotmail.com

**Keywords:** parasitoid wasps, venom, insect hosts, innate immunity, antimicrobial peptides

## Abstract

Insect host/parasitoid interactions are co-evolved systems in which host defenses are balanced by parasitoid mechanisms to disable or hide from host immune effectors. Here, we report that *Pteromalus puparum* venom impairs the antimicrobial activity of its host *Pieris rapae*. Inhibition zone results showed that bead injection induced the antimicrobial activity of the host hemolymph but that venom inhibited it. The cDNAs encoding cecropin and lysozyme were screened. Relative quantitative PCR results indicated that all of the microorganisms and bead injections up-regulated the transcript levels of the two genes but that venom down-regulated them. At 8 h post bead challenge, there was a peak in the transcript level of the cecropin gene, whereas the peak of lysozyme gene occurred at 24 h. The transcripts levels of the two genes were higher in the granulocytes and fat body than in other tissues. RNA interference decreased the transcript levels of the two genes and the antimicrobial activity of the pupal hemolymph. Venom injections similarly silenced the expression of the two genes during the first 8 h post-treatment in time- and dose-dependent manners, after which the silence effects abated. Additionally, recombinant cecropin and lysozyme had no significant effect on the emergence rate of pupae that were parasitized by *P. puparum* females. These findings suggest one mechanism of impairing host antimicrobial activity by parasitoid venom.

## 1. Introduction

At all developmental stages, most insects are actually and potentially subject to different infections by a broad range of foreign invaders, including viruses, bacteria, fungi, protozoans, and parasitoids [1,2]. Similar to other arthropods, insects lack an adaptive immune system and depend on the innate system to overcome invader infections [3]. Upon infection, insects activate their innate immune responses, which consist of cellular and humoral responses [4]. Many microorganisms or parasitoid eggs, which pass through the integument and physical barriers into the hemocoel of the insect hosts, quickly promote several types of insect immune responses [5,6,7]. The first step of insect immunity is non-self recognition, mediated by pattern recognition receptors (PRRs), to distinguish self from invaders based on the pathogen-associated molecular patterns that are present on their surfaces [8,9,10]. Cellular responses include phagocytosis, nodulation and encapsulation in the case of large invaders, such as the eggs of the parasitoid wasps. Cellular responses are characterized by interactions between hemocytes and invaders [11,12]. Humoral responses involve the induced biosynthesis of antimicrobial peptides (AMPs; e.g., cecropins) and enzymes (such as lysozyme and the enzymes that are involved in the activation of prophenoloxidase [PPO]) [13]. AMPs appear in the hemolymph of infected insects at 6–12 h post microorganism induction [14].

Endoparasitoid wasps complete their post-embryonic development within their hosts’ hemocoels, where they are vulnerable to host immunity, including cellular and humoral responses, such as encapsulation and melanization [15,16]. Oviposition into a host hemocoel triggers host immune responses. Non-permissive hosts effectively encapsulate and kill the parasitoid’s eggs. However, endoparasitoid wasps and their insect hosts have evolved mechanisms to evade or suppress their host immunity [17]. As a result of long-term co-evolution, some hymenopteran parasitoids possess both maternal and embryonic active factors to positively inhibit the defenses in their hosts [17,18]. These active factors include polydnaviruses (PDVs), virus-like particles contained in the ovary calyx fluid, proteins that are synthesized and secreted by the venom gland or ovaries, and teratocytes and their secreted proteins [17,19]. The roles of PDVs in impairing host immunity have received considerable attention [20,21]. For example, the *Hyposoter didymator* ichnovirus (HdIV) interferes with several aspects of host immune responses, such as regulating gene expression [22], impairing host encapsulation reaction [23], and suppressing PPO activation [24,25]. In addition to PDVs, the venom that is associated with parasitoid oviposition into the host is another virulence factor in the parasitoid/host system and functions in the disruption of host immunity. For instance, *Leptopilina boulardi* venom inhibits host immunity [26] due to the immunosuppressive factors in the venom Rho-GAP [27] and serpin [28], which suppress host hemocyte alteration and PPO activation, respectively. The extracellular superoxide dismutase that is isolated from the venom of the parasitoid *L. boulardi* can be used as a virulence factor to counteract the host humoral response [29]. The parasitoid wasp *Ganaspis* sp.1, venom SERCA (sarco/endoplasmic reticulum calcium ATPase) regulates *Drosophila* calcium levels and inhibits cellular immunity [30]. Generally, there is little knowledge on parasitoid venom influencing the antimicrobial activity of its host hemolymph, particularly at the molecular level.

The gregarious endoparasitoid wasp *Pteromalus puparum* (Hymenoptera: Pteromalidae) is a pupal parasitoid of *Pieris rapae* (cabbage white butterfly; Lepidoptera: Pieridae), which is a worldwide vegetable pest. This parasitoid injects venom but not PDVs into its host during its oviposition. *P. puparum* and its host *P. rapae* comprise an excellent model for studying the effects of venom on host physiology in the system not dependent on PDVs [31]. *P. puparum* venom alters the total number and morphology of host hemocytes [32]; impairs host cellular responses, including hemocyte spreading [33], phagocytosis, and encapsulation [34,35]; and decreases the melanization of the host hemolymph [36]. Furthermore, the component calreticulin, which was identified from *P. puparum* venom protein, suppresses host cellular responses by decreasing the expression levels of scavenger receptor and calreticulin genes [37]. However, whether *P. puparum* venom influences the antimicrobial activity of the host hemolymph and its mechanism are not completely understood.

Based on our subtractive-suppression hybridization study, we previously reported that the expression level of several host genes, including the *P. rapae* cecropin (Pr-cec) and lysozyme (Pr-lys) genes, is inhibited by treatment with venom from *P. puparum* [38]. Both cecropin and lysozyme play several roles in insect humoral responses, including antimicrobial activity [39], and melanization [40]. This paper reports experiments that were designed to test the hypothesis that *P. puparum* venom impairs the antimicrobial activity of the host hemolymph by decreasing the expression level of the genes encoding host cecropin and lysozyme. The outcomes of these experiments have provided novel insight into the mechanisms of parasitoid wasps regulating host humoral immunity.

## 2. Results

### 2.1. Induction and Inhibition of Antimicrobial Activity of Host Hemolymph

**Figure 1:**
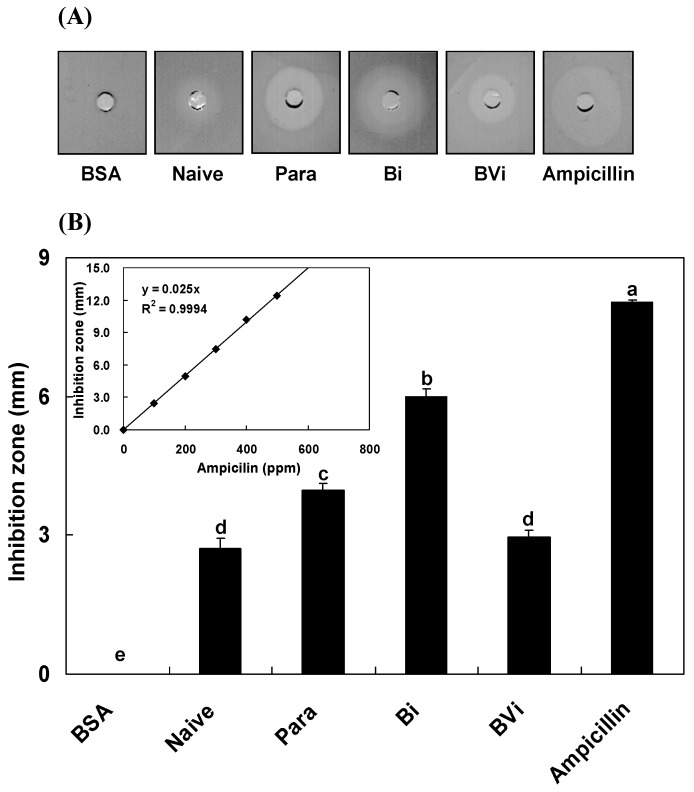
The influence of parasitization and venom treatments on the antimicrobial activity of *P. rapae* hemolymph. (**A**) The inhibition zone was represented by different treatments, including non-parasitization (naïve), parasitization (Para), bead-injection (Bi), and injection of beads + venom (BVi), while using BSA (350 ppm) and ampicillin (350 ppm) as negative and positive controls. Hemolymphs from differently treated pupae were isolated and collected 8 h post treatments. Each 10 µL of hemolymph from the treated pupae or bovine serum albumin (BSA) and ampicillin dilutions was applied into holes in the agarose plates. (**B**) Measurements of the inhibition zones among different treatments and controls. Different concentrations of ampicillin were used to construct a standard curve for reference (inset). Each treatment was replicated 5 times. Each value is represented as the mean ± SE. Different letters above the SE bars represent significant differences among the means at *P* = 0.05 after Bonferroni-correction for multiple testing.

Based on the results of our pre-experiments (data not shown), 8 h post treatment was the best sampling time for *P. rapae* pupae because the hemolymph that was collected from the treated pupae at this time period began to exhibit obviously antimicrobial activities and the diameter (dia.) of the inhibition zone of each treatment was different (Figure 1A). Using bovine serum albumin (BSA, 350 ppm) and ampicillin (350 ppm) for negative and positive controls, respectively, the results of the *in vitro* inhibition zone assay (Figure 1) showed that the BSA negative control presented almost no inhibition zone. Immunologically naïve host hemolymph only exhibited weak antimicrobial activity (inhibition zone dia. is 2.7 ± 0.2 mm, *n* = 5, mean ± standard error) for *Escherichia coli* compared to the ampicillin control, of which the inhibition zone dia. was large. Abiotic Sephadex A-50 beads were injected into the host hemocoel for mock parasitoid oviposition. The antimicrobial assay results (Figure 1B) demonstrated that the inhibition zone dia. of the host hemolymph was 4.0 ± 0.1 mm (*n* = 5) and 6.0 ± 0.2 mm (*n* = 5) post parasitization and bead injection for pupal hosts, respectively. The zone dia. of the host hemolymph after bead injection was larger than that post parasitization. The zone dia. of hemolymph that was extracted from pupae that were treated with the injection of beads plus venom was only 3.0 ± 0.2 mm (*n* = 5), which was not remarkably different from that of the immunologically naïve hemolymph according to the multiple comparison results (*F*_5, 24_ = 372.68, *P* < 0.0001 after Bonferroni-correction for multiple testing). These findings indicate that parasitization and bead injection induce the antimicrobial activity of the host hemolymph, while parasitoid venom treatment decreases this activity very significantly.

### 2.2. Molecular Cloning and Sequence Analyses of The Pr-cec and Pr-lys cDNAs

After screening the subtractive cDNA libraries of the *P. rapae* hemocytes and fat body, we obtained the full-length cDNA of the Pr-cec gene, which was 764 nucleotides long and contained a 192-nucleotide ORF encoding a 63-residue amino acid sequence that included a 24-residue predicted signal peptide (Supplementary Figure S1A). BLASTp results showed that the amino acid sequence of Pr-cec had a high level of identity (91%) with that of hinnavin II of *Artogeia rapae*. Multiple sequences alignment (Figure S1B) and phylogenetic analysis (Figure S2) results indicated that Pr-cec clustered with other lepidopteran cecropins and was different from those of the dipteran and nematode *Ascaris* cecropins (outgroup sequences). We also obtained the full-length cDNA of the Pr-lys gene after cDNA library screening; it was 610 nucleotides long and contained a 417-nucleotide ORF encoding a 138-residue amino acid sequence that included an 18-residue predicted signal peptide (Figure S3A). The BLASTp results showed that the amino acid sequence of Pr-lys had a high level of identity (98%) with that of lysozyme II of *A. rapae*. Multiple sequences alignment (Figure S3B) and phylogenetic analysis (Figure S4) results indicated that Pr-lys clustered with other lepidopteran lysozymes and was different from those of the dipteran and *Gallus gallus* (chick) lysozymes (outgroup sequences).

### 2.3. Effect of Immune Induction and Inhibition on Pr-cec and Pr-lys Gene Expression

**Figure 2:**
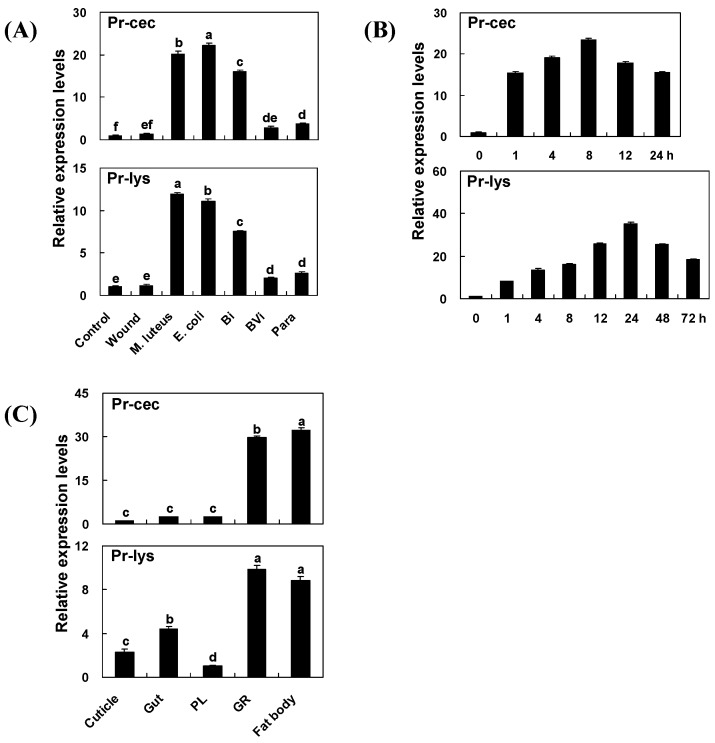
Expression profiles of Pr-cec and Pr-lys genes as performed by qPCR. (**A**) Different transcript levels of the Pr-cec and Pr-lys genes 8 h after immune induction or suppression, including non-treated (control), PBS-injection (wound), *M*. *luteus*-injection (M. luteus), *E*. *coli*-injection (E. coli), bead-injection (Bi), injection of bead + venom (BVi), and parasitization (Para). (**B**) Time courses of the Pr-cec and Pr-lys gene expression post bead injection. (**C**) Transcripts levels of the Pr-cec and Pr-lys genes in different tissues, including the cuticle, plasmatocyte (PL), granulocyte (GR), and fat body, 8 h post bead challenge. For qPCR, each treatment was replicated 5 times using *P*. *rapae* 18S rRNA gene as the internal control. The values are represented as the means ± SE. Different letters above the SE bars represent significant differences among the means at *P* = 0.05 after Bonferroni-correction for multiple testing.

Relative quantitative polymerase chain reaction (qPCR) was used to assay the expression level of the Pr-cec and Pr-lys genes in *P. rapae* pupae 8 h after immune challenge or suppression. Multiple comparison results (Figure 2A) showed that (for Pr-cec, *F*_6, 2__8_ = 486.62, *P* < 0.0001; for Pr-lys, *F*_6, 2__8_ = 715.14, *P* < 0.0001; after Bonferroni-correction for multiple testing) the Pr-cyc and Pr-lys gene transcripts levels were sharply up-regulated by injections of microorganisms (*Micrococcus luteus*, and *E. coli* K12) and inert beads (Sephadex A-50), and the immune inductions by *E. coli* and *M. luteus* caused the greatest increase in Pr-cec and Pr-lys mRNA levels, respectively. For the Pr-cec gene, the transcript level that was induced by *E*. *coli* injection was 22.3 and 16.2 times higher than that of the immunologically naïve and Pringle’s phosphate-buffered saline (PBS)-injected *P. rapae* pupae, respectively. For the Pr-lys gene, the transcript level that was induced by *M*. *luteus* injection was 11.9 and 10.7 times higher than that of immunologically naïve and PBS-injected *P. rapae* pupae, respectively. Post bead injection, the transcript level of the Pr-cec gene was 16.2 and 11.8 times higher than that of the immunologically naïve and PBS-injected *P. rapae* pupae, respectively, and was higher than that of the controls. Further, the transcript level of the Pr-lys gene was 7.5 and 6.8 times higher than that of the immunologically naïve and PBS-injected *P. rapae* pupae, respectively. The transcripts levels of the two genes as induced by bead injection were both weaker than those as induced by *E. coli* and *M. luteus*, respectively. In contrast, the levels of the Pr-cec and Pr-lys gene transcripts in pupae that were parasitized by *P. puparum* were only slightly higher than those in the two controls but significantly lower than those in both *E. coli-* and *M. luteus*-injected pupae. Additionally, the co-injection of venom + beads decreased the immune response due to bead injection, resulting in levels of Pr-cec and Pr-lys gene transcripts of only 0.16 and 0.25 of those given by beads induction (Figure 2A). Therefore, we infer that *P. puparum* venom suppresses the increased expression levels of the Pr-cec and Pr-lys genes.

### 2.4. Time Course of Immune Induction on Pr-cec and Pr-lys Gene Expression

Inert Sephadex beads were used to induce an immune response in *P. rapae* pupae, mimicking eggs of *P. puparum*, and the mRNA expression levels of Pr-cec and Pr-lys genes were measured at different sampling times post immune challenge. The qPCR results (Figure 2B) suggested that Pr-cec gene expression increased from 0 to 8 h after immune induction, with a peak of transcript accumulation at 4 h, 23.4 higher than that at 0 h. From 8 to 24 h post induction, the transcript level decreased to a level similar to that 1 h after treatment. For the Pr-lys gene (Figure 2B), the expression level increased from 0 to 24 h after immune challenge, with peak transcript accumulation at 24 h, 35.4 higher than that at 0 h. From 24 to 72 h post treatment, the mRNA expression level decreased to a level similar to that observed 8 h after treatment. The maximum transcript level of the Pr-lys gene appeared 24 h post bead injection, 16 h later than the appearance of the transcript peak of the Pr-cec gene. However, the trends of the expression of the two genes are similar.

### 2.5. Tissue Distribution of Pr-cec and Pr-lys Gene Expression

We recorded Pr-cec and Pr-lys gene expression 8 h after bead challenge in the cuticle, gut, plasmatocytes (PLs), granulocytes (GRs) and fat body, all of the tissues that were detected (Figure 2C). Multiple comparison results showed (for Pr-cec, *F*_4, 2__0_ = 1097.94, *P* < 0.0001; for Pr-lys, *F*_4, 2__0_ = 184.79, *P* < 0.0001; after Bonferroni-correction for multiple testing) that Pr-cec was expressed mostly in the fat body, 32.1-fold higher than in the cuticle, in which the mRNA expression level was lowest. The Pr-cec expression level in the GRs was 12.4 times higher than that in the PLs but still less than that in the fat body. The Pr-lys gene was expressed mostly in the GRs, 9.9-fold higher than in the PLs, in which the mRNA expression level was the lowest. This gene was also expressed in the cuticle and gut. The levels in these two tissues were 2.3 and 4.4 times higher than that in the PLs, respectively. For this gene, the transcript level in the fat body was less than that in the GRs.

### 2.6. Reduction of Antimicrobial Activity in the Host Hemolymph by the RNA Interference of Pr-cec and Pr-lys

**Figure 3:**
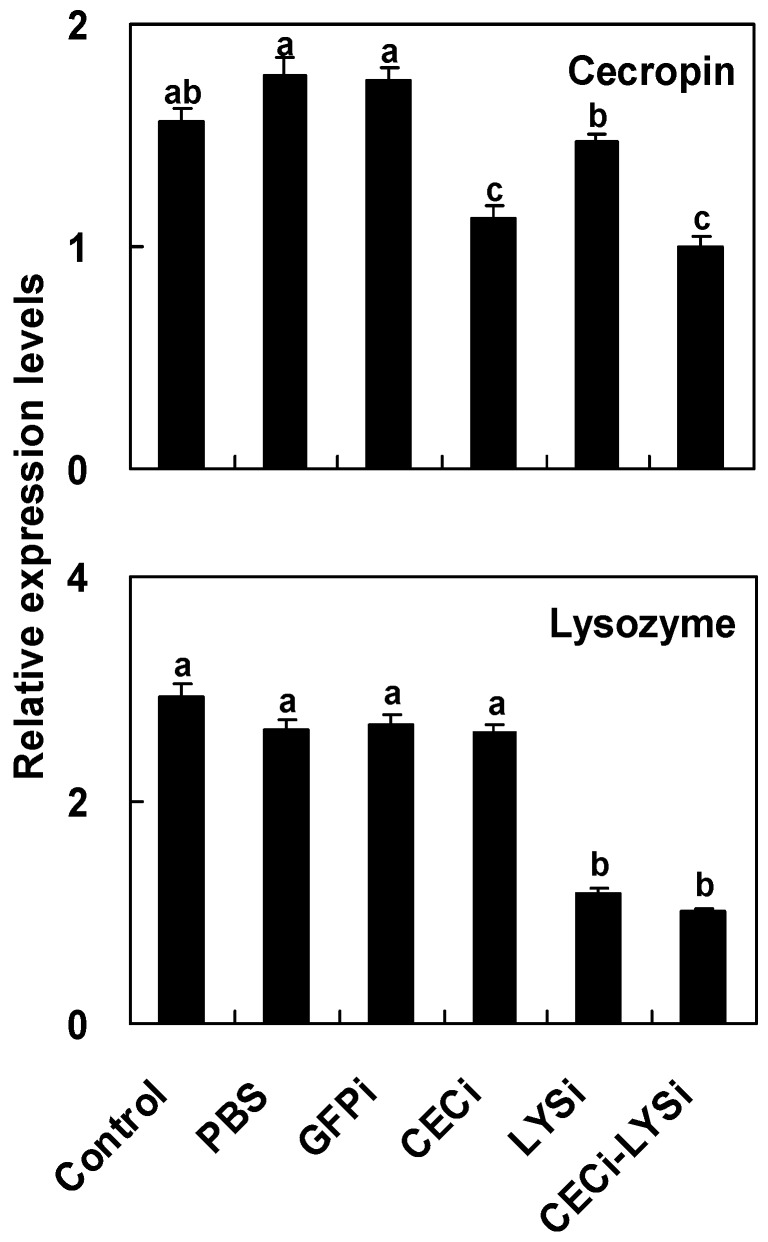
Suppressing the transcript levels of the Pr-cec and Pr-lys genes by dsRNA. To suppress the transcripts levels of the Pr-cec and Pr-lys genes, different dsRNA were immediately injected into host pupae post bead challenge, including dsRNA target to Pr-cec (CECi), Pr-lys (LYSi), and a combination of the dsRNA target to Pr-cec and to Pr-lys (CECi-LYSi). In parallel experiments, bead-injection pupae (control), bead-injection pupae that were treated with PBS (PBS), and those that were treated with the dsRNA that derived from green fluorescence protein sequence (GFPi) were used as the controls. The effects of RNA interference mediated by different dsRNA were confirmed by qPCR. For qPCR, each treatment was replicated 5 times. The values are represented as the means ± SE. Different letters above the SE bars represent significant differences among the means at *P* = 0.05 after Bonferroni-correction for multiple testing.

Multiple comparison results (Figure 3) showed that (for Pr-cec, *F*_5, 2__4_ = 30.38, *P* < 0.0001; for Pr-lys, *F*_5, 2__4_ = 104.49, *P* < 0.0001; after Bonferroni-correction for multiple testing) the transcripts levels of the Pr-cec and Pr-lys genes significantly decreased by approximately 50% and 67%, respectively, following double-strand RNA (dsRNA) treatment. The co-injection with the dsRNA target to both the Pr-cec (dscec) and Pr-lys (dslys) genes together decreased the transcript levels of both target genes. After RNA interference (RNAi), we measured the antimicrobial activities of the treated *P. rapae* pupae against *E*. *coli* and *M*. *luteus*. One-way ANOVA results (Table 1) showed that (for *E*. *coli*, *F*_5, 12_ = 18.17, *P* < 0.0001; for *M*. *luteus*, *F*_5, 12_ = 24.97, *P* < 0.0001; after Bonferroni-correction for multiple testing) the levels of the antimicrobial activities of pupal hemolymph against *E*. *coli* and *M*. *luteus* were lowest post co-injection with dscec and dslys. The dia. of the inhibition zones was 4.2 ± 0.2 and 7.1 ± 0.2 mm (*n* = 3), respectively. Post dscec injection, the antimicrobial activities of the pupal hemolymph were significantly impaired. The dia. of the inhibition zones against *E*. *coli* and *M*. *luteus* were 5.9 ± 0.3 and 10.5 ± 0.6 mm (*n* = 3), respectively, which were smaller than those of the controls. The same results occurred post dslys injection. The dia. of the inhibition zones against *E*. *coli* and *M*. *luteus* were 7.3 ± 0.2 and 7.8 ± 0.5 mm (*n* = 3), respectively. These results confirmed that decreasing the expression levels of Pr-cec and Pr-lys results in the impairment of the antimicrobial activity in the host hemolymph.

### 2.7. Time Course and Dose Effect of Venom Inhibition on Pr-cec and Pr-lys Gene Expression

**Figure 4:**
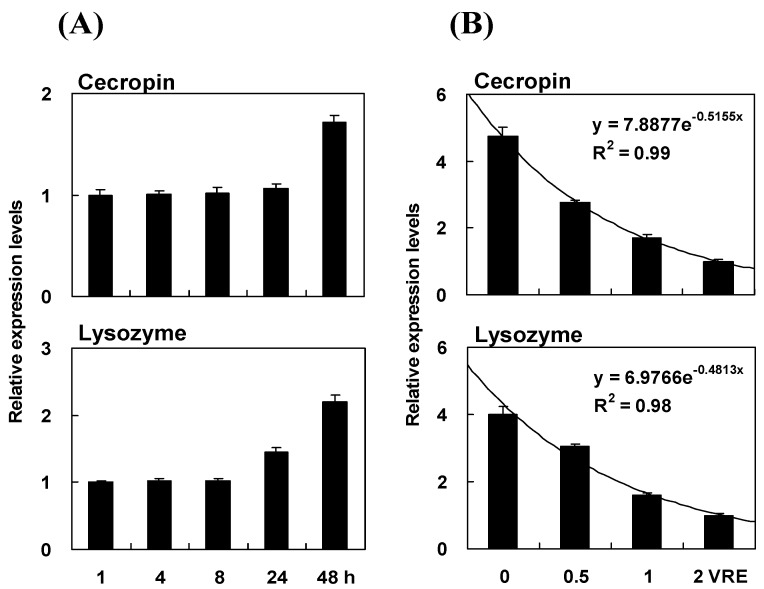
Influences of time (**A**) and venom dose (**B**) on the Pr-cec and Pr-lys transcript levels. (**A**) Time course of the transcript levels of the Pr-cec and Pr-lys genes at different sampling time periods following the venom + bead injection. (**B**) Dose effect of venom suppression on the transcript levels of the genes. The venom was collected from the female venom reservoir, and the extract was quantified as 2 venom reservoir equivalents (VREs)/µL. For time course experiments, 2 VREs were used for each time point. The *P*. *rapae* pupae were co-injected with beads plus venom at the same time. For qPCR, each treatment was replicated 3 times. The values are represented as the means ± SE.

The transcripts levels of the Pr-cec and Pr-lys genes remained low during the first 8 h following the bead + venom injections (Figure 4A). Panel A also shows the changes in the Pr-cec and Pr-lys gene expression during the 47 h post-treatment. The Pr-cec and Pr-lys gene expression levels increased significantly at 48 h post treatment (for *Pr-cec*, *F*_4, 1__5_ = 36.74, *P* < 0.0001; for *Pr-lys*, *F*_4, 1__5_ = 160.50, *P* < 0.0001; after Bonferroni-correction for multiple testing). For the Pr-cec gene, the transcript level at 48 h was 1.72-fold higher than that 1 h post treatment. For the Pr-lys gene, the mRNA expression level and transcript level at 48 h were 2.20-fold higher than that 1 h post treatment. Based on the results from Figure 4A, we infer that the inhibition effects of the venom on the mRNA expression of the two genes are much stronger during the first 8 h post treatments than those during 40 h following the bead + venom injections. Venom may protect the parasitoid offspring before 8 h after parasitization. After this time period, female parasitoid may possess other strategies to overcome the defenses from its host. The down-regulating influence of venom on Pr-cec and Pr-lys gene expression occurred in a dose-dependent manner (Figure 4B). By increasing the venom dosage from 0 to 2 venom reservoir equivalents (VREs), the decreasing effect on the host pupal gene expression was statistically significant and exponential (for Pr-cec, *R* = 0.99; for Pr-lys, *R* = 0.98). For 0 VRE injection, the mRNA expression levels of the two genes were highest. When the injection dosage was 0.5 VRE, the transcript levels of the Pr-cec and Pr-lys genes were 2.1 and 2.0 times higher than those observed when the dosage was 2.0 VREs, respectively.

### 2.8. Influence of Synthesized Pr-cec and Recombinant Pr-lys on the P. puparum Emergence Rate from the Parasitized Pupae

**Figure 5:**
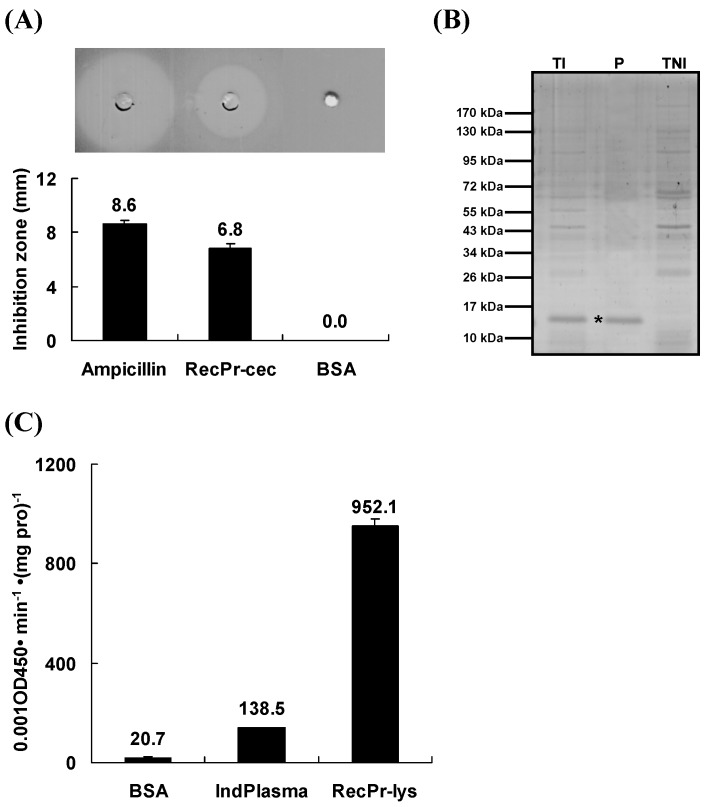
Activity assays for synthesized Pr-cec and recombinant Pr-lys. (**A**) Inhibition zone assay for synthesized Pr-cec (SynPr-cec) using ampicillin (350 ppm) as the positive control and 10 µg of BSA as the negative control. (**B**) An 15% SDS-PAGE analysis for recombinant Pr-lys (RecPr-lys) products under reducing conditions. “TI” represents the total protein (20 µg) of the induced *E*. *coli* Transetta (DE3) clone recombinantly expressing RecPr-lys. “P” indicates purified RecPr-lys (2 µg). “TNI” represents the total protein (20 µg) of the non-induced clone as described above. The asterisks represent the RecPr-lys band. (**C**) Absorbance assay for RecPr-lys, using *M*. *luteus* as the substrate, and bead-induced plasma (IndPlasma, with a total protein content of 10 µg) and BSA solution (10 µg) as the controls. Values are the means ± SE (*n* = 3).

**Figure 6:**
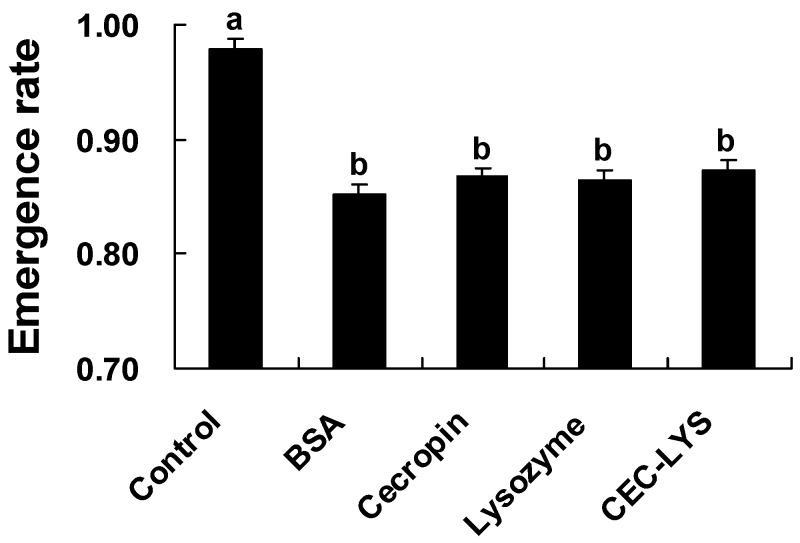
Influence of SynPr-cec and RecPr-lys on the emergence rate of the *P*. *puparum* offspring from the parasitized pupae. *P*. *rapae* pupae were first injected with different proteins, including BSA, SynPr-cec (Cecropin), RecPr-lys (Lysozyme) and a combination of SynPr-cec and RecPr-lys (CEC-LYS), using non-injected pupae as controls. Post injection, all of the treated or control pupae were parasitized *P. puparum*. Values are the mean ± SE (*n* = 20). All of the raw data were transformed by arcsine square root before a one-way ANOVA analysis. Histograms that are annotated with the same letter are not significantly different (*P* = 0.05 after Bonferroni-correction for multiple testing).

Using the solid phase method, the mature peptide of Pr-cec (SynPr-cec) was synthesized. The recombinant mature peptide of Pr-lys (RecPr-lys) was expressed by the *E. coli* system, after which the expression product that was fused with a 6× his-tag was purified, refolded and dialyzed to get the purely and actively recombinant product. Using inhibition zone and continuous spectrophotometric assays, the activities of SynPr-cec and RecPr-lys were measured. Figure 5A shows that SynPr-cec (10 µg) exhibited significantly antimicrobial activity. The dia. of the inhibition zone was 6.8 ± 0.3 mm (*n* = 3); its activity was similar to that of the ampicillin control (350 ppm, dia. of the zone is 8.6 ± 0.2 mm, *n* = 3). Panel B of Figure 5 indicates that the purification was successful, and the molecular mass on SDS-PAGE was approximately 15 kDa, close to the theoretical molecular weight (~14.5 kDa). The antimicrobial activity of RecPr-lys (10 µg) was 6.9-fold higher than that of the pupal hemolymph post bead induction, in which the content of the total protein was 10 µg. These results suggest that SynPr-cec and RecPr-lys were biologically active and able to function as AMPs. SynPr-cec and RecPr were first injected into *P. rapae* pupae, and then the injected pupae were parasitized by *P. puparum* under laboratory conditions. One-way ANOVA results (Figure 6) showed that (*F*_4, 95_ = 48.04, *P* < 0.0001) the emergence rate of *P. puparum* offspring from the non-treated pupae was highest (97.9 ± 0.8%, *n* = 20) compared to that of the other injected treatments. This result implies that the pupae were wounded post injection treatment, and this wound might reduce the emergence rate of *P. puparum* offspring. SynPr-cec and RecPr-lys injections (10 µg per pupa) did not significantly influence the emergence rate compared to that of the BSA injection control.

## 3. Discussion

The Hymenoptera is divided into several lineages, including herbivores and the monophyletic Apocrita [41]. In some views, the common ancestor of the Apocrita was a parasitoid, and outside of aculeates, all of the remaining apocritan groups consist predominantly or entirely of parasitoids [41]. All of the parasitoid wasps can be divided into externally feeding ectoparasitoids and internally feeding endoparasitoids. Indeed, endoparasitoid/host systems are always more complex than are the systems of ectoparasitoid/host because endoparasitoid wasps must maintain the viability of the hosts for a long period while simultaneously disabling immune defenses and manipulating growth [17,19].

The main response in lepidopteran larvae and pupae to parasitism is the formation of a melanized capsule that is composed of multiple layers of host hemocytes; the melanin that is formed may result in the death of the encapsulated offspring [15]. According to recent reports, the lepidopteran host promotes its cellular and humoral defenses to attack endoparasitoid infection [17]. These responses include hemocyte spreading and encapsulation, as well as hemolymph melanization. After oviposition, venom of the endoparasitoid *P. puparum* impairs the ability of *P. rapae* hemocytes to adhere to the surfaces of invaders, which diminishes the encapsulation of the eggs, allowing them to develop in host hemocoels [32]. Previously, we also reported that envenomation leads to a more global suppression of the gene expression related to host immune responses [38]. Then, the promotion of the host immune responses and the cellular reaction and melanization are impaired [35,36]. Similar results also have been discovered in other parasitoid/host systems, including *Microplitis demolitor*/*Pseudoplusia includens* [42,43,44], *Cotesia plutellae*/*Plutella xylostella* [18,45,46], and *L. boulardi* [47,48].

AMP induction, synthesis, and secretion are important aspects of insect humoral immunity, in addition to hemolymph melanization. AMPs play key roles in the defense and clearance of foreign pathogens [49]. Recent results have shown that the AMP levels in hosts that are parasitized by parasitoids or treated with maternal factors from the female adults are altered. Post parasitization by the parasitic wasps *in vivo*, the expression levels of the AMP genes are up-regulated [50]. In contrast, the expression levels of the AMP genes are suppressed in the hosts that are treated with parasitoid maternal factors *in vitro*, such as PDVs and venoms, which are isolated from the female adult parasitoids [51,52]. When parasitoid eggs enter the host hemocoel, they immediately trigger the host immunity, including cellular and humoral responses; of course, the expression levels of AMP genes are increased significantly. The active factors from female adult endoparasitoids impair host immunity to protect the offing living in the host hemocoel, involving the induction and synthesis of AMPs [53]. Here, we investigated the antimicrobial activities of the hemolymph from *P. rapae* hosts post different treatments. The results indicated that *P. puparum* parasitism induced the antimicrobial activity of host hemolymph; however, its level was much lower than that in the bead challenge, indicating that *P. puparum* venom, the key factor, impairs some AMPs that are expressed by host immune effectors, including hemocytes and fat body. The antimicrobial activity of the *P. rapae* host when injected with beads plus venom was similar to that of the immunologically naïve host. This result directly indicates that venom impairs AMP induction. We emphasize this point because impaired antimicrobial activity is typically associated with PDVs [54], which is not verified in *P. puparum* venom. The *P. puparum*/*P. rapae* relationship is not mediated by PDVs, and venom is the key factor of its female adults, which highlights the multipotentency of this venom.

To test our hypothesis, we cloned the full-length cDNAs of the Pr-cec and Pr-lys genes by screening the subtractive libraries of the host hemocytes and fat body. Pr-cec is a type of cecropin-like protein that is expressed by the *P. rapae* host. Cecropin is a type of antimicrobial peptide that has been discovered in different organisms, including insects [55], tunicates [56] and nematodes [57]. According to the results of a multiple sequences alignment and phylogenetic analysis, Pr-cec is clustered with the cecropin sequences of lepidopteran species and far from dipteran insects. Many cecropin precursors possess 62 to 64 amino acid residues [58], whereas Pr-cec contains 63 residues. The Gly residue at the C-terminus is assumed to be amidated by peptidylglycine α-amidating enzyme, probably to increase antimicrobial activity [59]. Several positive amino acid residues (“R” and “K”) of Pr-cec indicate that this peptide may bind to the negatively charged membranes of microorganisms to enhance its antimicrobial activity [60]. The antimicrobial activities of cecropins are related to their binding capability. These activities are usually composed of a strongly basic *N*-terminal region for binding to the microbial membranes and a long hydrophobic *C*-terminal stretch to form two short α-helices that facilitate the membrane-invasive activity [61]. Additionally, lysozyme is another important molecule in the innate immune system, including insect immunity. Pr-lys is a type of c-type lysozyme-like protein in the *P. rapae* host. For Pr-lys, its sequence is also clustered with lepidopteran species, and all lepidopteran lysozymes are clustered into the c-type lysozyme subgroup. However, the sequence of Pr-lys is different from that of dipteran lysozymes. The amino acid sequence identities among different insect lysozyme were very high, ranging from 40% to 80%. According to the results of the multiple sequences alignment, all of the lysozymes from different lepidopteran species are conserved, possessing all of the typical residues that are fundamental for three-dimensional structure and biological activity [62]. Pr-lys includes 8 cysteine residues and 2 catalytic sites of glutamic acid (Glu^31^) and aspartic acid (Asp^50^). Among the 8 cysteine residues, one is probably in the predicted signal peptide, whereas the other 7 residues are in the mature peptide of Pr-lys [63]. The disulfide bond bridges between pairs of cysteine residues stabilize the structures and functions of the lysozymes [62].

We evaluated the effects of different immune stimulations on the expression of the Pr-cec and Pr-lys genes by qPCR. The results indicate that gram-negative *E. coli* and gram-positive *M. luteus* strongly induce Pr-cec and Pr-lys gene expression, respectively. This result is similar to that in other insect species. For example, in *P. xylostella*, the transcripts levels of its cecropin Pxcec gene are significantly up-regulated by both gram-negative and gram-positive bacteria [52]. In *Ostrinia furnacalis*, the mRNA expression levels of its lysozyme OfLys6 are also increased remarkably by both gram-negative and gram-positive bacteria. Bead injection also induces Pr-cec and Pr-lys gene expression; however, the induction levels are weaker than those of *E. coli* or *M. luteus*. In addition, after latex bead injection, the cecropin and lysozyme genes were completely induced in the *S. frugiperda* hemocyte and fat body using a microarray approach [21]. Post venom treatment, the mRNA expression levels of Pr-cec and Pr-lys were significantly down-regulated. This result implies that *P. puparum* venom in particular inhibits the transcription of these two genes, which may be an important reason for the venom suppression of the antimicrobial activities of the host hemolymph. Our results are similar to those of a previous report [64]. However, there are still some differences between these results due to the sampling approaches (whole host body and only hemocytes, respectively) or time periods (1 h and 24 h post treatment, respectively). The qPCR results of the time course of the bead challenge (panel B of Figure 2) indicate that 1 h of the beads induction is able to trigger the related gene expression. The expression profiles of Pr-cec and Pr-lys as induced by immune challenge are very similar to the induced pattern of cecropin and lysozyme genes expression from other insect species [52,63,64]. In other species, the induced time for up-regulated gene expression was always 1 to 3 h post treatment. 

The hemocytes and fat body are the main immunity-conferring effectors in lepidopteran insects. Post bead injection, Pr-cec and Pr-lys were expressed in all of the measured tissues. For these two genes, the mRNA expression levels peaked in the GRs and fat body, respectively. The GRs and PLs are two important types of insect hemocytes; of these, GRs are generally more abundant than are PLs, strongly spread and attach to surfaces and provide the professional effect for cellular responses, including phagocytes and encapsulation, in lepidopteran insects [15]. According to our results, the GRs and fat body are the main effectors to highly express Pr-cec and Pr-lys, respectively. This result is the same as those from other insect species [52,62,64]. Additionally, our data show that silencing Pr-cec, Pr-lys and Pr-cec and Pr-lys gene expression together by dsRNA injection substantially impair antimicrobial activities by the host hemolymph. We infer from this result an observable biological function of the two genes in the process of antimicrobial in host humoral immunity, as also suggested by previous reports [39,40,65,66]. Using RNAi, the specific action of Pr-cec and Pr-lys cannot be easily identified because many genes are involved in host hemolymph antimicrobial actions. The gene expression and phenotype detections are measured 24 h post dsRNA injection. The results also tell us that dsRNA injection into the host *P. rapae* has effects after 24 h post treatments for the investigation of host gene function. This information is very help for future studies of *P. puparum*/*P. rapae* and other endoparasitoid/lepidopteran host systems.

Cecropin genes are induced by invader infections because there are several conserved regulating factors/motifs in the promoter regions of these genes. In *Drosophila*, the cecropin promoters contain a κB-like motif, GATA motif, and R1 motif, which are very important for the regulation of cecropin transcription and expression [67,68]. Similarly, these key motifs are also present in the gene promoters of lepidopteran cecropin genes [69]. However, in the sequence of the insect lysozyme gene promoter, there is also a κB-like motif [62,70], indicating that insect lysozyme genes can also be regulated by immune induction. PDVs and venom proteins carried by parasitoid female adults essentially suppress AMP gene expression in their hosts, including cecropin and lysozyme genes [25,52]. For PDVs, previous reports show that the genome of PDVs contains an IκB-like gene family [71]. The protein products that are expressed by these genes competitively bind to the NF-κB factors to form an irreversible complex [72] and inhibit the factor entering the nucleus of the host cell [52,73]. This inhibition cuts off NF-κB signaling transduction and impairs the translation and expression of AMP genes. For example, the *C. plutellae* bracovirus genome encodes eleven BEN family members, which shut down the antimicrobial activities of host hemolymph [46]. Other bracovirus ankyrin-repeat proteins differentially inhibit BmRelish1-dependent transcription in lepidopteran cells and impair the antimicrobial activities of these insect cells [74]. In the *Drosophila* system, PDV Ank proteins bind NF-κB homodimers and inhibit the processing of Relish [54]. These results imply that the effects of PDVs on insect host antimicrobial activity are much clearer than are those on venom components. Our recent results demonstrate that *P. puparum* venom impairs host antimicrobial activity by decreasing the expression level of Pr-cec and Pr-lys. However, the component or components that actually influence host humoral immunity have yet to be identified, requiring future research. 

Results from previous studies have shown that a high concentration of host AMP cannot kill or even attack a foreign invader possessing a large body shape, such as a parasitoid egg [52]. Our results also indicate that high concentrations SynPr-cec and RecPr-lys do not influence the emergence rate of the parasitoid offspring from the host pupa when parasitized by *P. puparum*, although these synthesized and recombinant AMPs are very effective in inhibiting microorganism growth. Then, the question becomes why does *P. puparum* impair host antimicrobial activity. A specific AMP level of the host is better for both host and parasitoid. For instance, larvae of the parasitoid wasp *Ampulex compressa* sanitize their host, the American cockroach, with a blend of antimicrobials [75]. Our hypothesis is that some endoparasitoids, including *P. puparum*, use their maternal factors such as venoms and PDVs to suppress host immunity, including via encapsulation and melanization, which play key roles in protecting their offspring in the host hemocoel. Host antimicrobial activity as one aspect of host immunity is also impaired non-specifically by parasitoid factors [76]. This result may be a negative effect on parasitism because the *P.* *rapae* pupae that are parasitized by *P. puparum* are always easily infected by pathogens. Whether the offspring of *P. puparum* can compensate for this negative influence remains unknown.

## 4. Experimental Section 

### 4.1. Insect Rearing

Cultures of *P*. *rapae* and *P*. *puparum* were maintained as previously described and used in all experiments [38]. After emerging, *P*. *puparum* females were collected and held in glass containers and fed *ad lib* on a 20% (*v*/*v*) honey solution to lengthen the life span for 3–4 days until the dissection of the venom reservoir and gland.

### 4.2. Crude Venom Preparation

Venom collection was described by Wu *et al*. [34] Five hundred glands and reservoirs were transferred to a sterilized 1.5-mL Eppendorf tube and centrifuged at 12,000 g for 20 min at 4 °C. The supernatant was collected and then filtered through a 0.22-µm cellulose acetate filter. The crude venom solution was diluted with PBS to a final concentration of 2 VREs/µL immediately before use.

### 4.3. Hemolymph Collection

Experimental preparations included parasitized host pupae, bead-injected pupae [50 Sephadex A-50 beads (GE Healthcare, Carlsbad, CA, USA) suspended in PBS using a sterilized 801 RN micro-syringe (Hamilton Bonaduz AG, Bonaduz, Switzerland)] and bead plus venom-injected pupae (2 VREs using a sterilized microsyringe). Immunologically naive host pupae were used as control preparations. Each treatment and corresponding control was repeated 5 times. Hemolymph was collected as previously described [38] 8 h post different treatments. Thirty microliters of the hemolymph were diluted with 470 µL of anticoagulant solution (0.9% NaCl, 0.942% KCl, 0.082% CaCl_2_, 2% EDTA). Diluted hemolymph was centrifuged at 12,000 g for 15 min at 8 °C, and the supernatant was collected and then filtered through a 0.22-µm cellulose acetate filter. The filtered solution was used directly or stored at −70 °C.

### 4.4. In Vitro Assay of Inhibition Zone

An inhibition zone assay was used to measure the antimicrobial activity of the collected host hemolymph according to the previously described approach [77]. *E. coli* K12 strain (Molecular Probe, Eugene, CA, USA) was used for the assay, and each Petri dish (dia. = 9.00 cm) contained 1 × 10^4^ bacteria. The antibiotic ampicillin (350 ppm) and BSA (350 ppm) were used as the negative and positive controls, respectively. The volume of each sample was 10 µL. Each treatment and corresponding control was repeated 5 times. The series of ampicillin concentrations was 0, 200, 400, 600 and 800 ppm, which were used to construct a standard curve [52] to calculate the relative value of the inhibition zones of each treatment. 

### 4.5. Obtained Full-length cDNA and Sequence Analysis

The full-length cDNAs of the Pr-cec and Pr-lys genes were screened from the subtractive cDNA libraries of the hemocyte and fat body, respectively [38]. The DNA Star software package (Version 5.02, Lasergene, Madison, WI, USA) was used to assemble the cDNA fragment sequence and to find the open reading frame (ORF) of full-length cDNA. A signal peptide was predicted by Signal P 4.1 program [78]. Sequence comparison and phylogenetic analysis were performed by MEGA version 5.1 software [79]. Sequences were aligned using Clustal W2 [80]. The tree was constructed by the unweighted pair grouping method with arithmetic mean (UPGMA), with statistical analysis by the bootstrap method using 1,000 replicates. The sequences that were used for the analyses are listed in Table S1.

### 4.6. Gene Expression Profile Analysis

The effects of immune induction and inhibition were measured on immunologically naive pupae of *P*. *rapae* (1 d after pupation). Pupae were exposed to parasitoid females to obtain parasitized hosts. In other experiments, pupae were injected with 5 × 10^4^ of *M*. *luteus* (Molecular Probe, Eugene, CA, USA) and *E*. *coli* K12, 50 Sephadex beads, and beads + venom (2VREs) suspended in 1 µL of sterilized PBS. Immunologically naïve and PBS-injected pupae were used as two controls. Each treatment or control was repeated 5 times. The total RNA samples were isolated from the pupae of each treatment and control using Trizol Reagent 8 h post treatment. The total RNA samples were treated with TURBO^™^ DNase (Ambion, Austin, TX, USA) to remove DNA contaminants. First-strand cDNA was synthesized using the SuperScript^™^ III First-Strand Synthesis System (Invitrogen, Shanghai, China), and random hexamers as primers. Each 10 µL of first-strand cDNA product was diluted with 190 µL of sterilized water before utilization. The qPCR experiments were carried out using the *P*. *rapae 18S rRNA* gene as an internal control as previously reported [35,56]. Primer pairs of Pr-cec, Pr-lys and 18S rRNA genes were designed using Primer3 [81] and are listed in Table 2. Each 25-µL reaction contained 12.5 µL of iQ TM SYBR^®^ Green Supermix (Bio-Rad, Shanghai, China), 1 µL of forward primer (200 nM), 1 µL of reverse primer (200 nM) and 10.5 µL of diluted cDNA. The thermal cycling conditions were 95 °C for 30 s, followed by 40 cycles of 95 °C for 5 s, 51 °C for 20 s, and 72 °C for 20 s. Amplification was monitored on an iCycler iQ^™^ Real-Time PCR Detection System (Bio-Rad). The specificity of the SYBR-Green PCR signal was further confirmed by melting curve analysis. The amplification efficiencies of the primers were tested. The experiments were repeated 5 times. The mRNA expression was quantified using the comparative cross-threshold method [82].

For the time course assay of changes in Pr-cec and Pr-lys gene expression in response to immune challenge, total RNA samples for Pr-cec measurement were isolated from the treated host pupae at 0, 1, 4, 8, 12 and 24 h post bead injection. For the Pr-lys gene, the sampling times were 0, 1, 4, 8, 12, 24, 48 and 72 h post beads injection. The experiments were repeated 5 times. The mRNA transcript levels of Pr-cec and Pr-lys were quantified using qPCR as described above.

For the time course assay of venom effects on Pr-cec and Pr-lys gene expression, 50 Sephadex beads plus venom (2 VREs) were injected into an immunologically naive pupa. To assay the dose effect, beads plus different doses of venom, including 0, 0.5, 1 and 2 VREs, were injected into an immunologically naïve pupa. The total RNA samples were isolated from the injected pupae at 1, 4, 8, 24 and 48 h post treatment for the time course and 8 h for the dose effect assays. The transcripts levels of the Pr-cec and Pr-lys genes in each total RNA sample were estimated as described above. 

To assess expression of Pr-cec and Pr-lys genes in different tissues, the treated host pupae were sampled, and the hemolymph was collected 8 h after bead injection as previously described [35,36]. After hemolymph collection, the cuticle, gut, and fat body were dissected. Plasmatocytes and granulocytes were separated using a modification of the method of Wiesner and Götz [83]. Briefly, 1 cm^3^ of loose nylon wool fiber (Wako, Tokyo, Japan) was inserted into a 5-mL sterilized syringe to plug the outlet. The inner wall of the syringe and the nylon wool fiber were washed repeatedly with anticoagulant solution, the syringe containing nylon wool fiber was vertically fixed on a steel frame and the outlet was sealed from the outside using a Parafilm membrane. One milliliter of hemolymph was slowly added to the fixed syringe. The hemolymph and the nylon wool fiber were co-incubated for 1 h at 28 °C to allow the granulocytes to adhere to the fiber firmly. The syringe outlet was then opened, and 10 mL of anticoagulant solution was poured through the syringe, collecting the eluted liquid. Most of the granulocytes were adsorbed on the fiber, while most of the plasmatocytes were in the eluted solution, which was centrifuged at 200 g for 10 min at 8 °C to prepare the plasmatocytes. In *P*. *rapae*, 97% of the hemocytes were plasmatocytes or granulocytes, and this method separated these two cell types with sufficient purity for subsequent experiments. The total RNA samples were isolated from all of the collected tissues as described above. The experiments were repeated 5 times. The mRNA transcript levels of the Pr-cec and Pr-lys genes were quantified using qPCR as previously described.

### 4.7. RNA Interference

dsRNA samples complementary to the Pr-cec, Pr-lys and green fluorescence protein (EGFP, control) genes were synthesized *in vitro* using the T7 RiboMAX^™^ Express RNAi System (Promega, Beijing, China) according to the manufacturer’s instructions and previously reported literature [35,36]. Primer pairs were designed for the Pr-cec, Pr-lys and EGFP genes (listed in Table 2). The pGEM^®^ T-Easy Vectors that inserted the Pr-cec and Pr-lys genes, as well as the pEGFP vector (Clontech, Mountain View, CA, USA) containing EGFP gene, were used as the plasmid DNA templates. Finally, treated dsRNA was purified, its integrity was confirmed by ethidium bromide gel staining, and its quantity was determined spectrophotometrically at A_260/280_.

Immunologically naive pupae (1 d after pupation) were first challenged by bead injection as described above. Post bead induction, the treated pupae then were injected with 20 µg of dsRNA in 2 μL of water for the Pr-cec and Pr-lys genes, the combination of the two dsRNAs for the Pr-cec and Pr-lys genes together (experimental), and the EGFP (control) gene (control). PBS injection controlled for the influence of PBS on mRNA expression. Each treatment or control was replicated 8 times:5 times for qPCR and 3 times for the inhibition zone assay.

For qPCR, the total RNA sample for each treated or control pupa was isolated, and the transcripts levels of the Pr-cec and Pr-lys genes were analyzed. PCR parameters, reference gene, quality control and the qPCR program for the Pr-cec and Pr-lys genes were identical to those in Section 4.6, and the primers are listed in Table 3. For the inhibition zone assay post dsRNA injection, hemolymph was collected from each treated or control pupa 8 h post treatment, and the antimicrobial activities were measured as described above.

### 4.8. Production of Synthesized Pr-cec and Recombinant Pr-lys

The peptide of Pr-cec that was used for the bioactivity assay in this manuscript was synthesized using a solid-phase peptide synthesis method; it was purified using Reverse phase HPLC, which was confirmed using MALDI-TOF mass spectrometry by the GenScript Company (Nanjing, China). The purity of SynPr-cec was greater than 90%. The purified SynPr-cec powder was used in subsequent experiments after dissolving in PBS.

RecPr-lys was recombinantly expressed using *E. coli* system. The cDNA fragment encoding mature Pr-SR protein was sub-cloned into a PET 32a (+) vector (Novagen, Shanghai, China) using specific sub-cloning primers containing SacI and SalI sites at the 5’ and 3’ ends, respectively (details shown in Table 2). PCR was performed as follows: 94 °C for 3 min; 30 cycles of 94 °C for 30 s, 51 °C for 30 s, and 72 °C for 30 s; and 72 °C for 5 min. The PCR product was digested with the SacI and SalI enzymes. The digested product was inserted into a SacI/SalI-digested PET 32a (+) vector and then transformed into *E*. *coli* Transetta (DE3) competent cells (TransGen, Beijing, China). The recombinant protein fused with His-tag was recombinantly expressed, purified and dialyzed according to a previous report [66]. Finally, the purified RcePr-lys was analyzed by 12% SDS-PAGE, and its concentration was determined by the Bradford method [84]. Using the inhibition zone assay as described above and continuous spectrophotometric assays [38], the activities of SynPr-cec and RecPr-lys were measured. Ampicillin (350 ppm), pupal host hemolymph induced by bead injection (the content of total protein was 10 µg), and BSA (10 µg) were used as the controls.

### 4.9. Emergence Rate Assay

Immunologically naïve pupae (1 d after pupation) were parasitized by *P. puparum* as mentioned above. At 2 h post successful parasitism, the parasitized pupae were injected with SynPr-cec (10 µg), RecPr-lys (10 µg), and a mixture of SynPr-cec (5 µg) and RecPr-lys (5 µg). The injection volume was 2 µL using both parasitized pupae without injection and pupae that were injected with BSA (10 µg) as controls. In this experiment, a total of 100 of pupae were used and divided into 5 groups. Each group contained 20 treated or control pupae, and each pupa was one replicate for the emergence rate calculation.

### 4.10. Statistical Analysis

Data in this manuscript was analyzed using the Data Processing System (DPS) software (version 9.50) [85]. All of the data were collected and evaluated before ANOVA and were fulfilled the criteria for applying this statistical analysis. Means were compared using the One-way ANOVA. The statistical significance was corrected by Bonferroni-correction for multiple testing and set at *P* = 0.05. Partial percentages data were first transformed to a quasi-normal distribution by arcsine-square root values prior to the statistical analysis.

## 5. Conclusions

The data that are reported in this paper support our original hypothesis set, in which *P. puparum* venom impairs the antimicrobial activity of host *P. rapae* by decreasing the transcript levels of the Pr-cec and Pr-lys genes. The following points apply. First, parasitization and venom treatment similarly impaired host antimicrobial activity compared to Sephadex bead challenge. Second, multiple sequences alignment and phylogenetic analysis results demonstrate the presence of both Pr-cec and Pr-lys in *P. rapae*, which clustered with lepidopteran cecropins and lysozymes, respectively. Third, while real and artificial invader infections increased Pr-cec and Pr-lys expression, venom treatments inhibited this expression in a time-dependent manner. Fourth, the highest Pr-cec and Pr-lys expression appeared in the granulocytes and fat body, respectively. Fifth, the anti-Pr-cec and anti-Pr-lys dsRNA treatments silenced the expression of these two genes and impaired the antimicrobial activity of the host hemolymph. Sixth, the influence of *P. puparum* venom was expressed in a time- and dose-dependent manner. Seventh, SynPr-cec and RecPr-lys possessed high biological activities and had no significant effect on the emergence rate of the parasitoid offspring from the parasitized pupae. In the future, why the parasitoid venom and which component/components reduce the antimicrobial activity of host hemolymph need to be further investigated.

## Figures and Tables

**Table 1:** Antimicrobial activities of the *P. rapae* pupal hemolymph against different microorganisms after RNA interference.

Microorganism	Treatments ^a^	Inhibition Zone (mm) ^b^
*Escherichia coli* (K12)	Control	7.8 ± 0.2 a
	PBS	8.2 ± 0.8 a
	GFPi	8.7 ± 0.3 a
	CECi	5.9 ± 0.3 bc
	LYSi	7.3 ± 0.2 ab
	CEC-LYSi	4.2 ± 0.2 c
*Micrococcus luteus*	Control	13.5 ± 0.7 a
	PBS	13.8 ± 0.6 a
	GFPi	13.3 ± 0.8 a
	CECi	10.5 ± 0.6 b
	LYSi	7.8 ± 0.5 c
	CEC-LYSi	7.1 ± 0.2 c

An aliquot of 10 µL of pupal hemolymph was used for the inhibition zone assay. The hemolymph was sampled 8 h post treatments. **^a^** Before control and RNAi treatment, the pupae were first injected with 50 beads to induce antimicrobial peptide gene expression. The pupae were non-injected (control) or injected with PBS (PBS), dsRNA derived from GFP (GFPi), Pr-cec (CECi), Pr-lys genes (LYSi), and a combination of the dsRNA target to Pr-cec and Pr-lys genes (CEC-LYSi) immediately post bead injection. Each dsRNA-treated pupa was injected with 20 µg of dsRNA (dissolved in 2 µL of RNase-free PBS), except for the CEC-LYSi treatment, with a total of 40 µg of dsRNA (20 µg of dscec and 20 µg of dslys dissolved in 4 µL of PBS). **^b^** Values are the means ± SE (*n* = 3). Different inhibition zones against the same microorganism were analyzed by a one-way ANOVA. The Values that are followed by different letters within the same microorganism are significantly different (*P* = 0.05 after Bonferroni-correction for multiple testing).

**Table 2:** PCR primers that were used for qPCR analysis and recombinant expression

Gene Names	Primer Sequences (from 5' to 3') ^a^		Functions
Pr-cec	SP	TTTCGCAACCACCTACAT	qPCR
	AP	TTCCAGCATTTCCATCAG	qPCR
Pr-lys	SP	TTGGGTATGTCTCGTTGAA	qPCR
	AP	TTGTGATGTCGTCCGTTGT	qPCR
18S rRNA	SP	TTTGCCTTATCAACTTTCG	qPCR
	AP	TGTGGTAGCCGTTTCTCA	qPCR
Pr-lys	Sub-SP	*TA*GAGCTCATGAAGTTAGCAGTATTCATTTTTG ^b^	expression
	Sub-AP	*AT*GTCGAC TTAACAAGAACTTATGTCAGGGAG ^b^	expression

**^a^** SP and AP are abbreviations for sense primers and anti-sense primers, respectively. **^b^** The extra bases upstream of the restriction site (underline) are the protective bases.

**Table 3:** Nucleotide sequences of the primers that were used in dsRNA synthesis

Primer Directions	Primer Names	Primer Sequences (from 5' to 3') ^a^
Forward	T7*Pr*CEC-F	*GGATCG*TAATACGACTCACTATAGGATGAATTTCGGAAAATTGTTTTTG
	*Pr*CEC-F	ATGAATTTCGGAAAATTGTTTTTG
	T7*Pr*LYS-F	*GGATCG*TAATACGACTCACTATAGGATGAAGTTAGCAGTATTCATTTTTG
	*Pr*LYS-F	ATGAAGTTAGCAGTATTCATTTTTG
	T7GFP-F	*GGATCG*TAATACGACTCACTATAGGAAGGGCGAGGAGCTGTTCACCG
	GFP-F	AAGGGCGAGGAGCTGTTCACCG
Reverse	T7*Pr*CEC-R	*GGATCG*TAATACGACTCACTATAGGCTATTTTCCTTTATAGATGGTGGCA
	*Pr*CEC-R	CTATTTTCCTTTATAGATGGTGGCA
	T7*Pr*LYS-R	*GGATCG*TAATACGACTCACTATAGGTTAACAAGAACTTATGTCAGGGAG
	*Pr*LYS-R	TTAACAAGAACTTATGTCAGGGAG
	T7GFP-R	*GGATCG*TAATACGACTCACTATAGGCAGCAGGACCATGTGATCGCGC
	GFP-R	CAGCAGGACCATGTGATCGCGC

**^a^** The extra bases upstream of the minimal T7 RNA polymerase promoter (underline) sequence may increase yield by allowing more efficient polymerase binding and initiation.

## Supplementary Materials

The following are available online at www.mdpi.com/2072-6651/8/2/52/s1.
