# Supplementary material for: Venom of Parasitoid Pteromalus puparum Impairs Host Humoral Antimicrobial Activity by Decreasing Host Cecropin and Lysozyme Gene Expression
Source: Toxins (Basel). 2016 Feb 20;8(2):52. doi: 10.3390/toxins8020052 (PMC4773805; doi:10.3390/toxins8020052)
Supplement: Supplementary file 1 [file toxins-08-00052-s001.pdf]

Supplementary Materials: Venom of Parasitoid *Pteromalus puparum* Impairs Host Humoral Antimicrobial Activity by Decreasing Host Cecropin and Lysozyme Gene Expression

Qi Fang, Bei-Bei Wang, Xin-Hai Ye, Fei Wang and Gong-Yin Ye

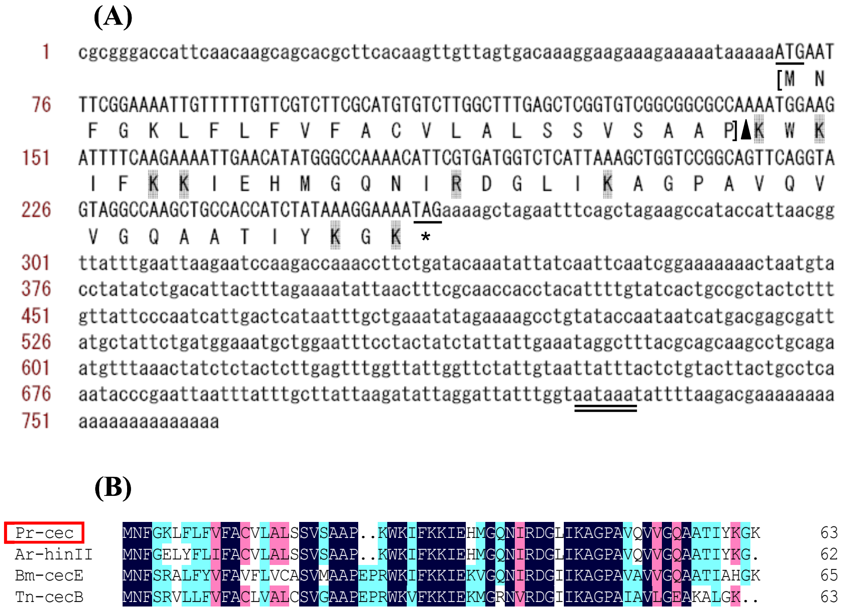

Figure S1. Nucleotide and deduced amino acid sequences of the Pr-cec gene. (**A**) Pr-cec cDNA (above) and amino acid (below) sequences are shown with a predicted signal peptide (shown in a pair of brackets), and the predicted cleavage site is indicated by a black triangle. Positively charged amino acid residues, arginine (K) and lysine (R), which bind to the negatively charged bacterial cell membrane, are shaded. Polyadenylation signal sequence is double underlined. Initial and stop codons are single underlined. (**B**) Multiple comparison of the amino acid sequence of Pr-cec with typical lepidopteran cecropin precursors (containing the signal peptide of each cecropin). The sequences that were used are listed in Supplementary Table S1. The boxes with the same color indicate the same amino acid residues in those species. Spots indicate gaps to optimize the sequence alignment. The target sequence is boxed in red and is the same in the other supplementary figures.

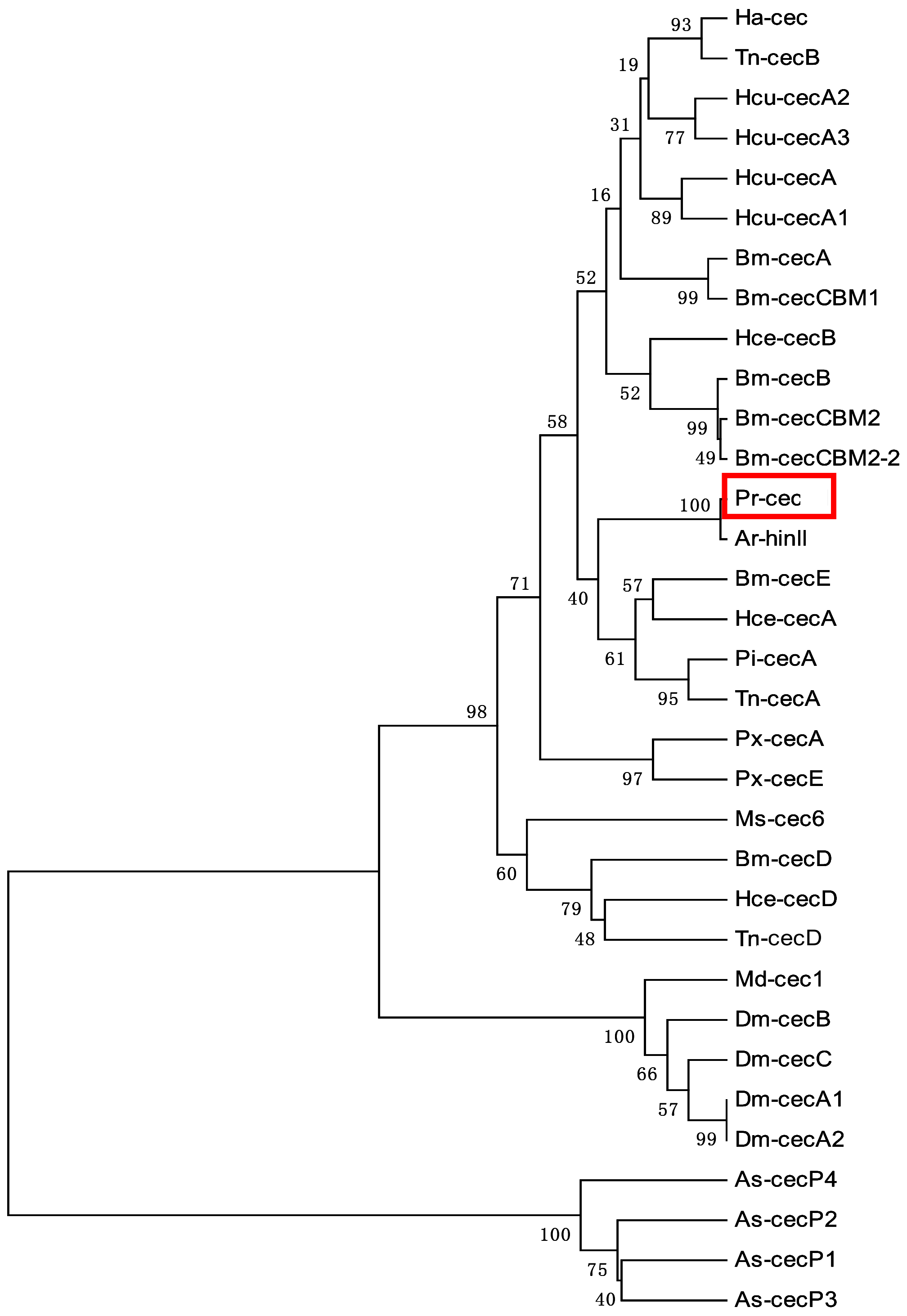

Figure S2. Phylogenetic analysis between Pr-cec and other cecropins. Construction was performed based on homology sequences that were calculated from the complete amino acid sequences of cecropin (cec) using the UPGMA method. Sequences were selected from NCBI databases (listed in Supplementary Table S1), and the sequence of the nematode *Ascaris* cecropins (AscecP1-4) was used as the out-group. The values in the tree are bootstrap support values.

(A)

```
1    gcggggatacgcctttggtcaagagtagagaacacaactATGAAGTTAGCAGTATTCATTTTTGCACTTGCTGCT
                                            [M  K  L  A  V  F  I  F  A  L  A  A
76   CTGTTCGGAGCAGAAGCCGTTACGTTTACAAGATGCCAATTGGTGCGCGAATTAAGGAATCAAGGCTTTCCAGAA
      L  F  G  A  E  A]▲V  T  F  T  R  C  Q  L  V  R  E  L  R  N  Q  G  F  P  E
151  ACTAAAATGAGAGATTGGGTATGTCTCGTTGAAAACGAGAGTAGCCATAACACAGCCAAAGTGGGAAAAGTGAAC
      T  K  M  R  D  W  V  C  L  V  E  N  E  S  S  H  N  T  A  K  V  G  K  V  N
226  AAGAATGGTTCCAGAGACTACGGTCTCTTCCAGATCAACGACAAGTACTGGTGCAGCAATACTAACACTGCCGGA
      K  N  G  S  R  D  Y  G  L  F  Q  I  N  D  K  Y  W  C  S  N  T  N  T  A  G
301  AAAGACTGCAATGTCACATGTGCGCAGGTGACAACGGACGACATCACAAAAGCTGCAACCTGTGCTAAAAAGATC
      K  D  C  N  V  T  C  A  Q  V  T  T  D  D  I  T  K  A  A  T  C  A  K  K  I
376  TTTAAGCGCCATGGATTCAATGCTTGGTATGGTTGGAAGAACCACTGCCAAGGCTCTCTCCCTGACATAAGTTCT
      F  K  R  H  G  F  N  A  W  Y  G  W  K  N  H  C  Q  G  S  L  P  D  I  S  S
451  TGTTAAaagctctactctagttaaacaatattatataaaacactacgatcctatacagaatattgtgaaatataata
      C  ·  *
526  tatctatgtaattagttatttcacaaatgtttctcaaataaaaataattaaatccaaaaaaaaaaaaaaaaaa
```

(B)

```
Pi-lys  MQKLTVFLLAVAALCLSCEAKYFKTNCELVHELRRQGFPEDKMRDWVCLIQNESGRNTSKMGTINKNGSRDYGLFQINDK   80
Sl-lys  MQKLTVFVLALAAICLHCEAKQFTR.CGLVQELRRQGFPEDKMRDWVCLVENESGRKTDKTGTVNKNGSRDYGLFQINDK   79
Ms-lys  MYKLVIF..ALFAFAYHSEAKHFSR.CELVHELRRQGFPENLMRDWVCLVENESSRYTDKVGRVNKNGSRDYGLFQINDK   77
Pr-lys  .MKLAVFIFALAAL.FGAEAVTFTR.CQLVRELRNQGFPETKMRDWVCLVENESSHNTAKVGKVNKNGSRDYGLFQINDK   77
Ar-lys  .MKLAVFIFALAAL.FGAEAVTFTR.CQLVRELRNQGFPETKMRDWVCLVENESSRNTAKVGKVNKNGSRDYGLFQINDK   77

Pi-lys  YWCSKTSTPGKDCNVTCADMLLDDITKASTCAKKIYKRHKFQAWYGWRNHCQG.ALPDISKC   141
Sl-lys  YWCSNTSTPGKDCSVTCAQMLLDDITLASQCAKKIYKRHKFEAWYGWKNHCKGKTLPDISNC   141
Ms-lys  YWCSNGSTPGKDCNVKCSDLLIDDITKASTCAKKIYKRHKFQAWYGWRNHCQG.SLPDISSC   138
Pr-lys  YWCSNTNTAGKDCNVTCAQVTTDDITKAATCAKKIFKRHGFNAWYGWKNHCQG.SLPDISSC   138
Ar-lys  YWCSNTNTAGKDCNVTCAQVTTDDITKAATCAKKIFKRHGFNAWYGWRNHCQG.SLPDISSC   138
```

Figure S3. Nucleotide and deduced amino acid sequences of the Pr-lys gene. (**A**) Pr-lys cDNA (above) and amino acid (below) sequences are shown with a predicted signal peptide (shown in a pair of brackets), and the predicted cleavage site is indicated by a black triangle. The polyadenylation signal sequence is doubly underlined. Initial and stop codons are single underlined. (**B**) Multiple comparison of the amino acid sequence of Pr-lys with typical lepidopteran lysozyme precursors (containing the signal peptide of each lysozyme). The sequences that were used are listed in Supplementary Table S1. The boxes with the same color indicate the same amino acid residues in those species. Spots indicate gaps to optimize the sequence alignment.

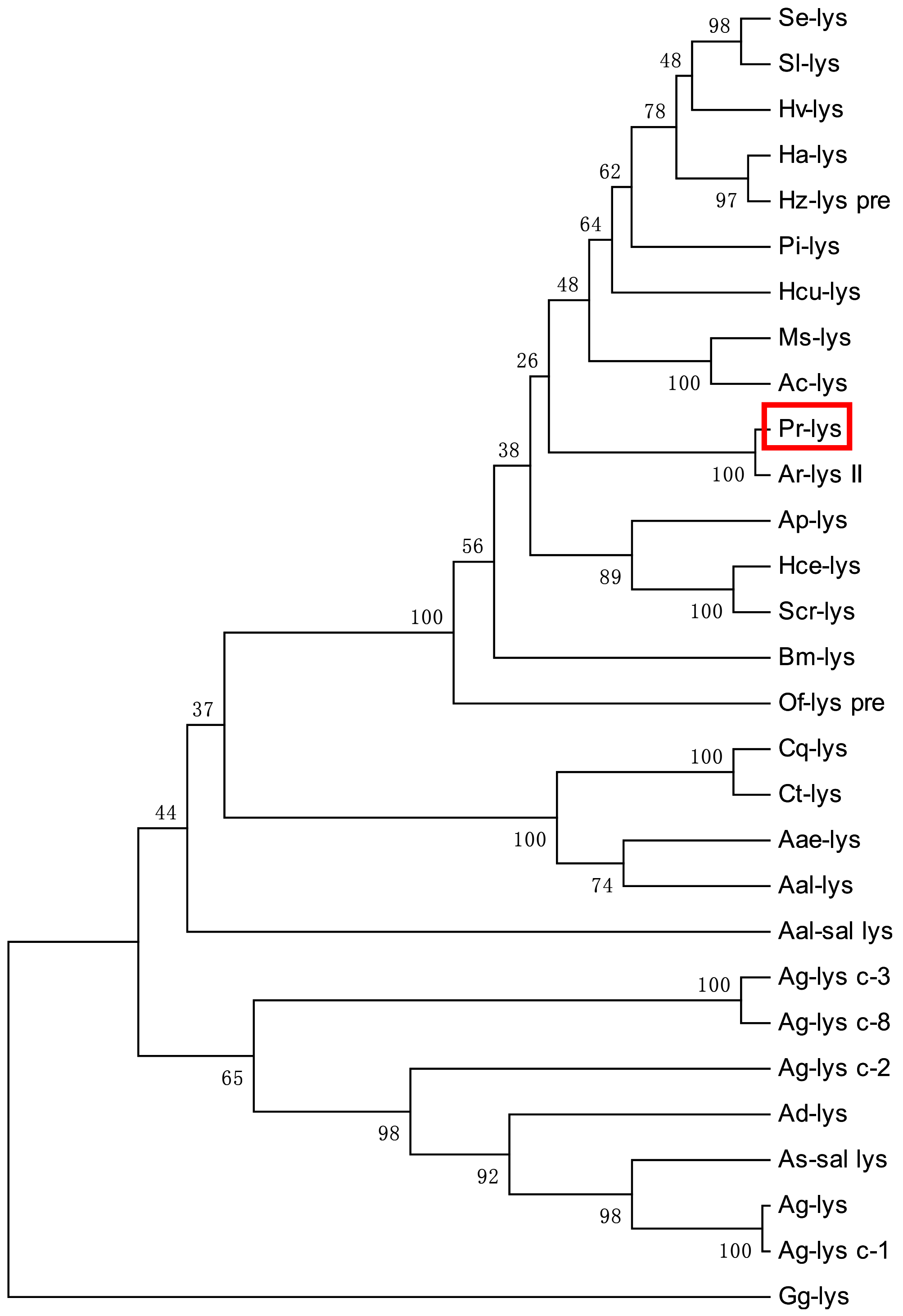

Figure S4. Phylogenetic analysis between Pr-lys and other lysozymes. Construction was performed based on homology sequences that were calculated from the complete amino acid sequences of lysozyme (lys) using the UPGMA method. Sequences were selected from NCBI databases (listed in Supplementary Table S1). The sequence of the lysozyme of *Gallus gallus* (Chicken) (Gg-lys) was used as the out-group. The values in the tree are bootstrap support values.

Table S1. Deduced amino acid sequences of cecropin and lysozyme, which were used in the multiple sequences alignments and phylogenetic tree constructions.

**Sequences of Cecropin**					
**Name**	**Accession No.**	**Species**	**Name**	**Accession No.**	**Species**
Pr-cec	-	*Pieris rapae*	Bm-cecCBM1	NP_001037030	*Bombyx mori*
Ar-hinII	AAT94287	*Artogeia rapae*	Bm-cecCBM2	NP_001037031	*Bombyx mori*
As-cecP1	BAD89085	*Ascaris suum*	Bm-cecCBM2-2	NP_001037032	*Bombyx mori*
As-cecP2	BAD89086	*Ascaris suum*	Bm-cecD	NP_001036833	*Bombyx mori*
As-cecP3	BAD89091	*Ascaris suum*	Bm-cecE	NP_001037392	*Bombyx mori*
As-cecP4	BAD89092	*Ascaris suum*	Dm-cecA1	NP_524588	*Drosophila melanogaster*
Bm-cecA	NP_001037462	*Bombyx mori*	Dm-cecA2	NP_524589	*Drosophila melanogaster*
Bm-cecB	NP_001037460	*Bombyx mori*	Dm-cecB	BAA28722	*Drosophila melanogaster*
Hce-cecA	P01507	*Hyalophora cecropia*	Dm-cecC	AAB82507	*Drosophila melanogaster*
Hce-cecB	P01508	*Hyalophora cecropia*	Md-cec1	AAL08023	*Musca domestica*
Hce-cecD	P01510	*Hyalophora cecropia*	Ms-cec6	CAL25128	*Manduca sexta*
Tn-cecA	P50724	*Trichoplusia ni*	Pi-cecA	AAR99379	*Pseudoplusia includens*
Tn-cecB	ABV68872	*Trichoplusia ni*	Px-cecA	BAF64473	*Plutella xylostella*
Tn-cecD	ABV68873	*Trichoplusia ni*	Px-cecE	BAF36816	*Plutella xylostella*
Hcu-cecA	P50720	*Hyphantria cunea*	Hcu-cecA2	P50722	*Hyphantria cunea*
Hcu-cecA1	P50721	*Hyphantria cunea*	Hcu-cecA3	P50723	*Hyphantria cunea*
Ha-cec	AAX51304	*Helicoverpa armigera*	-	-	-
**Sequences of lysozyme**					
**Name**	**Accession No.**	**Species**	**Name**	**Accession No.**	**Species**
Pr-lys	-	*Pieris rapae*	Ha-lys	ABF51015	*Helicoverpa armigera*
Hcu-lys	AAA84747	*Hyphantria cunea*	Ac-lys	AAN87265	*Agrius convolvuli*
Ct-lys	ACJ64375	*Culex tarsalis*	Aae-lys	AAU09087	*Aedes aegypti*
Ag-lys	AAC47326	*Anopheles gambiae*	Hz-lys pre	ACL51928	*Helicoverpa zea*
Ap-lys	ABC73705	*Antheraea pernyi*	Hce-lys	P05105	*Hyalophora cecropia*
Ar-lysII	AAT94286	*Artogeia rapae*	Hv-lys	AAD00078	*Heliothis virescens*
Bm-lys	NP_001037448	*Bombyx mori*	Ms-lys	AAB31190	*Manduca sexta*
Gg-lys	NP_990612	*Gallus gallus* (Chicken)	Of-lys pre	ABN54797	*Ostrinia furnacalis*
Cq-lys	XP_001847112	*Culex quinquefasciatus*	Pi-lys	AAS48094	*Pseudoplusia includens*
Aal-lys	AAM11885	*Aedes albopictus*	Scr-lys	BAB20806	*Samia cynthia ricini*
Ag-lys c-2	AAT51797	*Anopheles gambiae*	Se-lys	AAP03061	*Spodoptera exigua*
Ag-lys c-8	AAY21241	*Anopheles gambiae*	Sl-lys	ACI16106	*Spodoptera litura*
As-sal lys	AAO74844	*Anopheles stephensi*	Aal-sal lys	AAV90643	*Aedes albopictus*
Ad-lys	ACI30031	*Anopheles darlingi*	Ag-lys c-3	AAT51798	*Anopheles gambiae*
Ag-lys c-1	AAY24699	*Anopheles gambiae*	-	-	-
